# Association between thyroid disorders and COVID-19: a protocol for a systematic review and meta-analysis

**DOI:** 10.1186/s13044-021-00113-1

**Published:** 2021-09-29

**Authors:** Soraya Doustmohammadian, Azam Doustmohammadian, Marjan Momeni

**Affiliations:** 1 School of Medical Sciences, Semnan University of Medical Sciences, Semnan, Iran; 2 Gastrointestinal and Liver Diseases Research Center, Iran University of Medical Sciences, Behafarin St., Karimkhan Ave., Vali-asr Sq, Tehran, Iran; 3 Department of Health Information Technology, School of Allied Medical Sciences, Semnan University of Medical Sciences, Semnan, Iran

**Keywords:** Thyroid disorders, Protocol, Systematic, COVID-19

## Abstract

**Background:**

The novel coronavirus (COVID-19) epidemic initially appeared in Wuhan, Hubei Province, China, on 31 December 2019 and was spread rapidly worldwide. Most underlying diseases reported with COVID-19 patients are diabetes, hypertension, coronary heart diseases, and cerebrovascular disease. We do not know whether individuals with thyroid disease are at increased risk of COVID-19 infection.

**Methods:**

Two experienced researchers will conduct an electronic search of the databases including PubMed/MEDLINE, the Cochrane Reviews, and the Cochrane Central Register of Controlled Trials (CENTRAL), Web of Science, Scopus, and ProQuest, for articles published since October 2019. Clinical trials and observational studies will be included. Studies will be screened after de-duplication. A standardized data extraction form will be developed through discussions with the review team and will be revised after piloting. An appropriate risk of bias assessment tool will be used to assess the quality of studies. Two independent reviewers will assess the eligibility, extraction of detailed information, and quality assessment of studies. The results will be pooled for meta-analysis, subgroup analysis and/or descriptive analysis based on the included data conditions.

**Conclusion:**

Results of this study will provide current evidence on the association of COVID-19 diseases with any thyroid disorders such as hypothyroidism, thyrotoxicosis, and thyroid cancer with or without radioiodine therapy. Findings will be disseminated in peer-reviewed publications and conference presentations.

**Trial registration:**

PROSPERO registration number: CRD42020184289.

https://www.crd.york.ac.uk/PROSPERO/#recordDetails

**Supplementary Information:**

The online version contains supplementary material available at 10.1186/s13044-021-00113-1.

## Introduction

### Description of the condition

The novel coronavirus (COVID-19) epidemic initially appeared in Wuhan, Hubei Province, China, on 31 December 2019 and was spread rapidly worldwide [1]. As of 13 August 2021, the total number of 206,263,033 cases of COVID-19 have been reported, including 4,348,337 deaths, the majority of which from the United State of America (636298) and India (430285) [2].

The virus can be transmitted to healthy people through the small respiratory droplets of COVID 19 during coughing or exhaling. Fever, dyspnea, and dry cough are the most common symptoms of COVID-19. Usually, these symptoms are mild and begin gradually. About 80% of patients recover from the disease without the need for specific treatment. But the elderly or those with underlying diseases such as hypertension, cardiac problems or diabetes, chronic obstructive airway disease, progress to more severe illnesses [3].

Most underlying diseases reported with COVID-19 patients are diabetes, hypertension, coronary heart diseases, and cerebrovascular disease [4]. Both diabetes types 1 and 2 can increase the risk of contraction of COVID-19 due to immune dysfunction [5]. Amid the underlying diseases, thyroid disorders association with COVID-19 remains mainly unclear [6].

### Description and function of intervention

Some pathophysiological scenarios have suggested that thyroid hormones may bind to the integrin receptors on the cell membrane, thus, activating signal pathways inside the cell and regulating the transcription of genes involved in anti-apoptotic, angiogenetic properties, and ultimately, supporting cell proliferation [7]. On the other hand, hypothyroidism is a remediable cause of hypertension and significantly impacts lipid profile and cardiovascular disease. Since thyroid disorder is associated with high blood pressure, dyslipidemia, and cardiovascular disease, hypothyroid patients may be at higher risk for COVID-19 infection. Being on the suppressive dose of levothyroxine may increase the expression of integrin αvβ3 on the cell surface; therefore, T4 may enhance to a more extent SARS-CoV-2 internalization, possibly worsening the prognosis in case of COVID-19 [8]. It seems that patients taking anti-thyroid drugs (ATDs) are not at higher risk for COVID-19 or developing more severe disease, unless in neutropenia due to ATDs, which is very rare [9]. Patients on corticosteroids for thyroid eye disease may be at high risk of severe illness from COVID-19 and need to take more precautions [5].

Patients, who have previously received treatment for thyroid cancer, such as surgery, or radioiodine therapy, are not known at higher risk for COVID-19 infection [5]. For patients with thyroid cancer categorized as ‘low risk’, surgery may be postponed until a safer time depending on several factors, including the number of COVID-19 patients locally and staff availability. Radioiodine therapy in most cases of thyroid cancer is not urgent and may be safely delayed. However, these hypotheses should be confirmed before making conclusions.

### Why the review is important

So far, no definitive treatment has been found for COVID-19 infection, and unfortunately, the incidence of this disease and its mortality rate is rising in the world. Therefore, further identification and control of related underlying diseases will be effective in preventing COVID-19 infection or preventing the progression of severe infection, or reduce the mortality rate.

A growing number of published observational studies have examined whether thyroid disorders increase the risk of adverse outcomes in patients with COVID-19 [10]. In a retrospective study from New York City, the results showed that preexisting hypothyroidism does not affect the prognosis of COVID-19 [11]. Furthermore, some narrative and systematic reviews investigated the relationship between thyroid and COVID-19 and reported contradictory results [12, 13]. Some researchers in a narrative review concluded that thyroid diseases seem not to affect the progression of COVID-19 [13]. A meta-analysis of observational studies reported worse outcomes among COVID-19 patients with preexisting thyroid disorders [14]; however, several limitations may have impacted the result, including study selection methods and a limited number of cases.

Thus, thyroid disorder as an underlying disease affecting worse outcomes in COVID-19 patients remains fully un-clarified. The current study aims to systematically assess all the available evidence, and if possible, a meta-analysis on the potential association of thyroid diseases with worse outcomes in COVID-19 patients.

## Methods

### Protocol and registration

Guided by the Cochrane Collaboration Handbook of Systematic Reviews [15], the Preferred Reporting Items for Systematic Review and Meta-Analysis Statement [16], and Protocols Extension (PRISMA-P) [17]. The recommended items in the Preferred Reporting Items for Systematic Review and Meta-Analysis Protocols (PRISMA-P) checklist were completed (Additional file 1), the protocol design, search strategy, synthesis, and reporting of findings from this systematic review will be presented. The protocol was registered at the International Prospective Register of Systematic Reviews (PROSPERO) under registration number CRD42020184289.

### Criteria for considering studies for this review

#### Types of studies

We will include observational studies, including case-control and cohort studies in any language that evaluate the association of COVID-19 diseases with any thyroid disorders such as hypothyroidism, thyrotoxicosis, and thyroid cancer with or without radioiodine therapy. Articles in other languages will be translated using Google Translate and/or deep translate: https://www.deepl.com/translator.

#### Types of participants

We will include studies in which human adult patients (≥ 18 years old) who have a laboratory-confirmed diagnosis of COVID-19 with information on thyroid disease and clinical outcome of the clinically validated definition of mortality, severe COVID-19, ARDS, ICU admission, and disease progression will be considered in this review.

#### Types of comparison/exposure

In this systematic review, thyroid disorders such as hypothyroidism and hyperthyroidism with or without treatment (being euthyroid or not) and thyroid cancer with or without radioiodine therapy are evaluated as factors affecting the clinical outcomes and prognosis COVID − 19 disease.

#### Types of outcome measures

The primary outcome is a poor composite outcome comprising mortality, severe COVID-19, ARDS (Acute Respiratory Distress Syndrome), duration of hospitalization, need for ICU admission, and disease progression. ARDS is defined as per the World Health Organization (WHO) temporary guidance of Severe Acute Respiratory Infection (SARI) of COVID-19, including the acute onset, chest imaging, and origin of pulmonary infiltrates, and oxygenation impairment [18]. Severe COVID-19 is defined as patients who have any of the following features at the time of admission or after admission: (1) respiratory distress (≥ 30 breaths/min); (2) oxygen saturation at rest ≤93%; (3) ratio of partial pressure of arterial oxygen (PaO2) to the fractional concentration of oxygen inspired air (fiO2) 300 mmHg; or (4) critical complication (respiratory failure, septic shock, and or multiple organ failure) [19].

According to exclusion criteria reported in Table 1, irrelevant study design and study population, as well as observational studies that examined risk factors for adverse clinical outcomes in patients infected by other coronaviruses or the association between Covid-19 and non-thyroidal illness or sub-acute thyroiditis, will be excluded.

**Table 1:** Study eligibility and exclusion criteria

Inclusion criteria	
**Design**	All observational studies such as case-control and cohort studies
**Participants**	All adult patients (≥ 18 years old) who have a laboratory-confirmed diagnosis of COVID-19 with information on thyroid disease (any thyroid disorders such as hypothyroidism and hyperthyroidism with or without treatment (being euthyroid or not) and thyroid cancer with or without radioiodine therapy)
**Comparison/exposure**	Thyroid disorders such as hypothyroidism and hyperthyroidism with or without treatment (being euthyroid or not) and thyroid cancer with or without radioiodine therapy are evaluated as factors affecting the clinical outcomes and prognosis COVID −19 disease.
**Outcomes**	The outcome of interest will be any clinical outcomes in patients with COVID-19, including mortality, severe COVID-19, ARDS, duration of hospitalization, need for ICU care/ admission to ICU, and disease progression
**Exclusion criteria**	•. irrelevant study design, including review articles, letters, commentaries or case reports and case series with COVID-19 patients below 20 •. observational studies that examined risk factors for adverse clinical outcomes in patients infected by other coronaviruses (e.g., Severe Acute Respiratory Syndrome and Middle East Respiratory Syndrome) •. observational studies on the association between Covid-19 and non-thyroidal illness or sub-acute thyroiditis •. irrelevant study population (< 18 years old)

### Search methods for identification of studies

#### Information sources and search strategy

In order to do a comprehensive search, both official and non-official sources will be searched by two researchers. A search strategy was created using a combination of MeSH terms and keywords. The search strategy will be adapted for each database (Table 1). Electronic databases including PubMed/MEDLINE, the Cochrane Reviews, and the Cochrane Central Register of Controlled Trials (CENTRAL), Web of Science, Scopus, and ProQuest will be searched from October 2019. In addition, clinical trial registries, including Clinicaltrial.gov, will be searched for ongoing trials. Also, grey literature (e.g., thesis or dissertations, conference papers, and final reports of research projects) will be searched in the ProQuest for related thesis/ dissertation, SCOPUS, and web of science for conference papers. The search process will be conducted using various Boolean operators (AND/OR), truncation, wildcards, etc., either individually or in combination..

For the database, we will use the suggested query or the strategy suited to the environment of the database to search the studies. The primary search strategy has been developed in PubMed as the main database in medical sciences by using different words for both concepts thyroid and COVID-19 and the MeSH database. After that, a search strategy will be adopted for other databases. No language limitation will be considered.

### Data collection and analysis

#### Screening and selection processes

**Fig 1:**
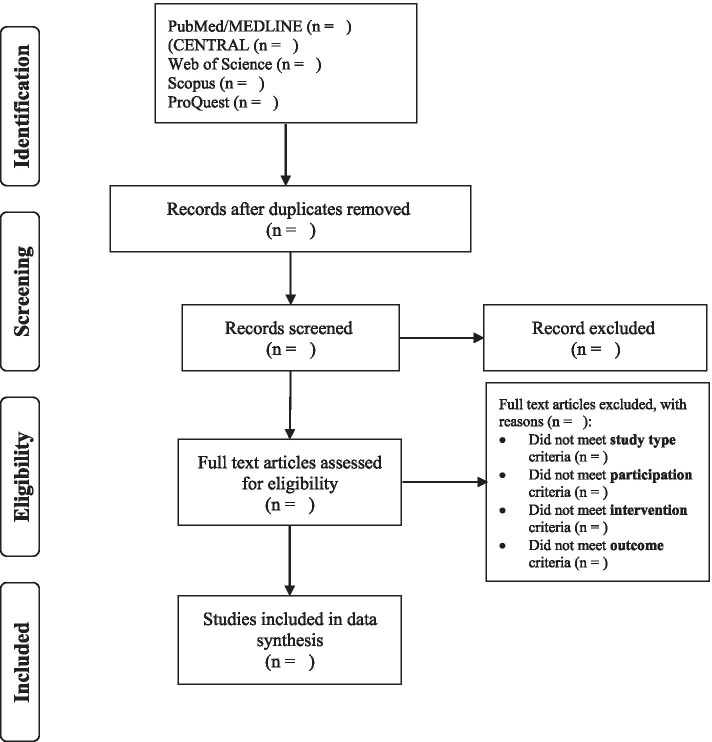
PRISMA diagram

All Search results will be imported to Endnote reference manager software to store and organize them for further evaluation. First, duplicates will be detected and deleted, and the remaining articles will be merged into one library. Second, two reviewers independently will screen the remaining studies for eligibility by their titles and abstracts. The resolution of any conflicts will be made by a third reviewer or by discussion. A full-text screening will be carried out according to inclusion and exclusion criteria. The reasons for the exclusion of the full-text article will be documented. The PRISMA (preferred reporting items for systematic review and met analyses) flowchart (Fig. 1) [20] will be utilized to document the selection process. A table of ‘Characteristics of excluded studies’ will be designed as well.

#### Data extraction and management

Data extraction will be performed using a standardized form including the following data fields:

Study characteristics including study title, author(s), publication year, geographic origin, study design. Participant characteristics including sample size, age (e.g., mean with standard deviation, range), and gender. Outcome results, including the primary outcome, comprise mortality, severe COVID-19, ARDS, duration of hospitalization, need for ICU admission, and disease progression.

The standardized form will be pilot tested to ensure that data is accurately and consistently captured.

#### Assessment of risk of bias (quality) in included studies

Two reviewers will independently assess the risk of bias, including selection bias, performance bias, detection bias, attrition bias and reporting bias, by assigning a rating of low, high or unclear risk of bias. Quality assessment of included studies will be evaluated using the Newcastle-Ottawa quality assessment scale (NOS) [21]. The total score of the scale is nine stars, and > seven stars are considered high quality. The data extraction and quality assessment will be performed independently by two reviewers then will be checked by a third reviewer. Disagreements will be resolved by discussion.

The overall strength of the evidence will be illustrated in a risk of bias graph. Forest plots provide the overall effect. Effect sizes will be calculated for each individual study, as well.

#### Dealing with missing data

To address the missing data, researchers will contact to original authors via email. If data remain unavailable after two attempts, we will first analyze without these data, followed by a sensitivity analysis assuming that the missing data had adverse outcomes. These studies with missing data will also be included in the systematic review, where a table for the findings of each study and a narrative synthesis will be presented. Attrition rates, including drop-outs, losses to follow up, and withdrawals, will be investigated.

#### Assessment of heterogeneity

A Chi-square test will be used for heterogeneity assessment, with an alpha of 0.05 used for statistical significance and the I^2^ test [22]. I^2^ values of 25, 50, and 75% correspond to low, medium, and high levels of heterogeneity.

#### Assessment of publication bias

Funnel plots for asymmetry Egger’s test [23] and Begg’s test [24] will be adopted to detect the publication bias. The results will be considered to indicate potential minor study effects when *P* values are <.10.

#### Data synthesis

We will perform meta-analyses to compute the odds ratio (OR) and 95% CI of thyroid diseases in COVID-19 patients with or without severe illness and non-survivors or survivors. Adjusted odds ratios based on correction for comorbidity, age, gender, smoking, body mass index and other potential confounders will be presented. The meta-analyses will be performed using the inverse variance method with the random-effects model. The statistical heterogeneity will be assessed with the I^2^ statistic, and values of < 25%, 26–50, and > 50% are considered low, moderate, and high level of heterogeneity, respectively. To assess the small-study effect, we will perform a regression-based Harbord’s test for the dichotomous outcome. Begg’s funnel-plot analysis will be performed to assess the risk of publication bias.

If significant heterogeneity is detected, we will present data in a narrative style rather than meta-analysis, and the findings will be summarized and discussed. Conclusions will also be drawn based on the power of each of the studies.

All analyses will be conducted using STATA (13.0; Stata Corporation, College Station, Texas, USA Stata), and the statistical significance level is set at *P* < .05.

#### Additional analyses

We will conduct a subgroup analysis for each component of the poor composite outcome. Sensitivity analyses will be conducted by excluding studies with a sample size of less than 100. Random-effects meta-regression will be performed using a restricted-maximum likelihood for pre-specified variables including age, gender, hypertension, diabetes mellitus, cardiovascular disease, and chronic obstructive pulmonary disease (COPD).

## Discussion

This systematic review and meta-analysis will be the first of its kind in explaining the relationship between thyroid disease and COVID-19 infection. It appears that COVID-19 disease expresses more serious in patients with comorbid disease such as diabetes mellitus, hypertension, cardiovascular disease. It is unclear whether thyroid diseases patients are at higher risk of severe covid-19 infection or mortality.

The potential limitation at the study level is the lack of sufficient research to evaluate the association between thyroid disorders and COVID-19. This review is intended to publish in a peer-reviewed journal. Analyses and scripts will be made publicly available, and any changes to the protocol will be documented. All actions of this review are based on the Cochrane Collaboration Handbook of Systematic Reviews to give compelling evidence and better guide in clinic practice.

## Conclusion

This study will provide current evidence on the association of COVID-19 diseases with thyroid disorders such as hypothyroidism, thyrotoxicosis, and thyroid cancer with or without radioiodine therapy. Findings will be disseminated in peer-reviewed publications and conference presentations.

## Supplementary Information

**Additional file 1.**

## Data Availability

Data sharing is not applicable to this article as no datasets were generated or analyzed during the current study.

## Abbreviations

ARDS: Acute respiratory distress syndrome
ATD: Anti thyroid drug
COPD: Chronic obstructive pulmonary disease
PTC: Papillary thyroid cancer
FTC: Follicular thyroid cancer
MNG: Multi nodular goiter
PCR: Polymerase chain reaction
PROBLAST: Prediction model Risk Of Bias ASsessment Tool
QUADAS: Quality Assessment of Diagnostic Accuracy Studies
TPO: Thyroid peroxidase antibody
ROBINS-I: Risk of Bias in Non-randomized Studies of Intervention
SARI: Severe acute respiratory infection
Tg: Thyroglobulin
WHO: World Health Organization
